# ROMA Index Is an Effective Predictor for Advanced Endometrial Cancer before Surgery

**DOI:** 10.1155/2022/7409368

**Published:** 2022-12-23

**Authors:** Jianzhang Wang, Ping Xu, Gen Zou, Meichen Yin, Xinqi Mao, Xinmei Zhang

**Affiliations:** Department of Gynecology, Women's Hospital, School of Medicine, Zhejiang University, Hangzhou 310006, China

## Abstract

There is a high rate of inconformity between clinical staging and surgical-pathologic staging in endometrial cancer. Many patients with advanced endometrial cancer are preoperatively understaged and thereby do not receive the optimal therapy. Here, we aimed to develop a predictive model or biomarker for preoperative diagnosis of advanced endometrial cancer via multivariate logistic regression analysis. In this study, 259 eligible patients were included, and 195 patients were assigned to the training dataset and 64 patients to validation dataset. Age, menopause status, sterilization situation, parity, body mass index, hypertension, diabetes mellitus, tumor size, and ovarian malignancy algorithm (ROMA) index were included as predictive variables, and the binary outcome was advanced endometrial cancer or not. When the *P* value was set as less than 0.01 in forward stepwise regression, only ROMA index was retained. The odds ratio of being positive ROMA index was 15.531 times that of negative value. The area under receiver operating characteristic curve was 0.790 in the training dataset and 0.776 in the validation dataset. The decision curve analysis curve showed that the prediction by ROMA index added more net benefits for almost all threshold probabilities. Therefore, ROMA index is an effective predictor for advanced endometrial cancer before surgery. Since ROMA index is a standard, measurable, and reliable laboratory test, it can be used as a reference tool for gynecologists to design the appropriate therapeutic schedule for patients with high-stage endometrial cancer before surgery.

## 1. Introduction

In developed countries, endometrial cancer is the most common gynecological malignancy, and its incidence is still on the rise globally [1]. Thanks to the symptom of postmenopausal bleeding, most patients can be diagnosed at an early stage (limited to the uterus) [2]. Surgery is the mainstay of the initial management of this disease, and total hysterectomy and bilateral salpingo-oophorectomy is the basic operation [3]. According to the International Federation of Gynecology and Obstetrics (FIGO) 2009 guidelines, endometrial cancer is divided into four stages (stage I, stage II, stage III, and stage IV). Primary advanced endometrial cancer (stage III and stage IV) was diagnosed in approximately 10 to 15% of patients [4], and the 5-year survival rate has been reported to be only about 17% [5]. The less common advanced disease account for roughly half of endometrial cancer related deaths [6]. Given this, advanced endometrial cancer deserves more attention in the future study.

It is reported that the improved outcomes for the stage III-IV patients if the surgery area was sufficient and no residual lesions were left [7, 8]. However, it is difficult to know the exact stage preoperatively in clinical practice because the FIGO staging is based on pathological evaluation after surgery, which may cause insufficient scope of operation especially for advanced endometrial cancer. It is reported to be inaccurate for clinical staging in reflecting actual disease extent, which causes understaging in 15-20% of patients [9, 10]. Therefore, accurate preoperative assessment of stage is important for every patient especially for the fertility-sparing women and inoperable patients. In addition, there are many other situations such as the following. Firstly, neoadjuvant chemotherapy significantly improved the overall survival for advanced-stage patients before surgery [11]. Secondly, sentinel lymph node mapping is not suitable for the advanced endometrial cancer. Thirdly, the arrangement of staff and time depends on the complexity of surgery. Therefore, we are better to know more about stage information because early detection of the advanced endometrial cancer is quite important for the optimal treatment planning. Currently, many methods have been applied for the stage prediction before surgery such as ultrasound, computed tomography (CT), magnetic resonance imaging (MRI), and biomarkers, while the results are usually not ideal as expected [12]. Therefore, the novel and simple method should be further investigated.

Epidemiological studies have found that some risk factors may correlate to endometrial cancer such as estrogen therapy, old age, race, family history, obesity, infertility, and radiotherapy and that higher percentage of patients suffer from hypertension and diabetes mellitus. However, the association between risk factors and the progression of endometrial cancer depends on the probability of individuals, and the disease may become advanced without symptoms. Here, we selected accessible and stable factors including age, menopause status, sterilization situation, parity, body mass index (BMI), hypertension, diabetes, tumor size, ovarian malignancy algorithm (ROMA) index, and surgical procedures to develop a comprehensive prediction model for the advanced endometrial cancer.

## 2. Materials and Methods

### 2.1. Study Design

This project was a retrospective study of endometrial cancer registered in Women's Hospital, School of Medicine, Zhejiang University. We collected hospitalized endometrial cancer patients with surgery from January 2020 to June 2022. This cohort study was conducted in accordance with the Declaration of Helsinki and obtained approval from the hospital's Ethics Committee (IRB-20220290-R). The sample was divided into two groups. Specifically, 75% of patients were randomly assigned to the training dataset, and 25% of patients were in validation dataset.

### 2.2. Study Subjects

All study subjects were endometrial cancer patients who did the surgery in hospital. Furthermore, the patients must meet the following inclusion criteria: (1) the pathologic diagnosis was endometrial cancer including all histological types; (2) patients with complete medical records that could be acquired from the electronic medical records system including age, menopause status, sterilization situation, parity, BMI, hypertension, diabetes mellitus, tumor size, ROMA index, and surgical procedures; (3) surgical pathologic stage was confirmed by the specialists based on the FIGO 2009 guidelines; (4) patients did not meet the exclusion criteria. The exclusion criteria included the following terms: (1) patients who also suffer from other malignant tumors; (2) patients with acute infection; (3) patients with endometriosis and adenomyosis; (4) patients with radiotherapy or chemotherapy before surgery.

### 2.3. Study Variables: Predictors

The predictive variables included age, menopause status, sterilization situation, parity, BMI, hypertension, diabetes mellitus, tumor size, and ROMA index. Among them, age and tumor size were continuous variables, and the others were dichotomous variables. Age, menopause status, sterilization situation, parity, BMI, hypertension, and diabetes mellitus were always well recorded in the electronic medical record. Tumor size refers to the maximum diameter of the tumor measured by ultrasound or MRI. The cutoffs of ROMA index were 11.40% in premenopausal women and 29.90% in postmenopausal patients, respectively.

### 2.4. Study Variables: Primary Outcome

The primary outcome was the surgical pathologic stages of every eligible patients evaluated by at least two specialists after the staging operation. According to the FIGO 2009 guidelines, endometrial cancer can be divided into stage I, stage II, stage III, and stage IV. Patients with stage I and stage II were included in the early stage, which was defined as the value of 0. Patients with stage III and stage IV were included in advanced stage, which was assigned the value of 1. Therefore, the primary outcome was a dichotomous variable.

### 2.5. Statistical Analysis

The training dataset was used to develop the prediction model using a logistic regression model, and the validation dataset was applied for evaluating the predictive performance. Variables that were significant in the univariate analysis or meaningful in the clinical practice were included in the multivariate regression. Statistical analysis was conducted using STATA 15.0 for Windows. Forward stepwise selection was applied in the multivariable logistic regression analysis when the likelihood ratio test with Akaike's information criterion (AIC) was performed as the stopping rule [13]. The area under the receiver operating characteristic (ROC) curve was applied for the quantification of the discrimination. Decision curve analysis (DCA) was conducted to quantify the net benefits at different threshold probabilities in the dataset [14]. All statistical tests were two sided, and *P* value of < 0.05 was considered significant.

## 3. Results

### 3.1. Patient Characteristics

According to the inclusion and exclusion criteria, 259 patients were finally included in this study. Among them, 195 patients were assigned to the training dataset, and 64 patients were to validation dataset. A small portion of patients was suffered from advanced endometrial cancer (stage = 1 in Table 1) (14.9% of the training cohort and 18.8% of the validation cohort). In the training data, 14.5% of early-stage patients were positive for ROMA index, while 72.4% of advanced-stage patients were positive for ROMA index. In the validation data, ROMA index was positive in 12% of early-stage patients and in 67% of advanced endometrial patients. The characteristics of patients were summarized in Table 1, and the demographics in two datasets were comparable.

### 3.2. ROMA Index and Tumor Size Were Predictive Factors for Advanced Endometrial Cancer before Surgery in the Training Data

In the training data including 195 patients, age, menopause status, sterilization situation, parity, BMI, hypertension, diabetes mellitus, tumor size, and ROMA index were included as predictive variables, and the binary outcome was stages III-IV endometrial cancer (the value = 1) or not (the value = 0). When factors for which *P* < 0.05 in the multivariate logistic regression analysis were preliminarily considered as significant, ROMA index and tumor size were selected as the best subset of predictors for the probability of advanced endometrial cancer. The result showed that the odds ratio (OR) of being positive ROMA index was 5.167 times that of negative value, and tumor size was another risk factor for advanced endometrial cancer (OR = 1.644) (Table 2). The above results demonstrated that ROMA index and tumor size were predictive factors for advanced endometrial cancer before surgery.

### 3.3. The Combination of ROMA Index and Tumor Size Effectively Predicted Advanced Endometrial Cancer before Surgery

**Figure 1:**
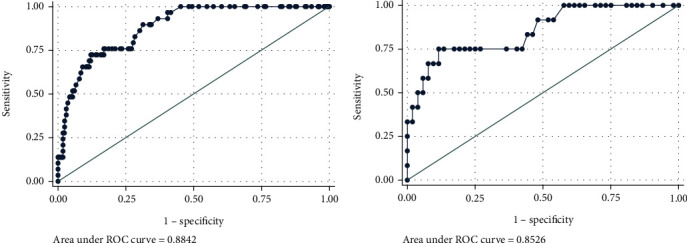
Receiver operating characteristic (ROC) curve for the preoperative prediction of advanced endometrial cancer by the combination of ROMA and tumor size. (a) The area under ROC was 0.884 in the training dataset. (b) The area under ROC was 0.853 in the validating dataset.

**Figure 2:**
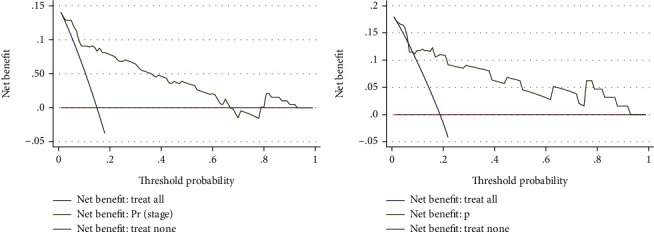
Decision curve analysis (DCA) curve of the prediction of advanced endometrial cancer before surgery by the combination of ROMA and tumor size. (a) The attainable net benefit in the training dataset. (b) The attainable net benefit in the validating dataset.

In the training data, the receiver operating characteristic (ROC) curve was used to perform preoperative prediction via combination of ROMA index and tumor size, and the results showed that the area under curve (AUC) was 0.884 (Figure 1(a), Table 2), indicating that the included risk factors could effectively predict advanced endometrial cancer before surgery. To further validate the predictive ability of ROMA index and tumor size, 64 patients were used as the training data. *P* value was generated using the following formula calculated from the training data, *P* = 1/(1 + exp(−(−4.098 + 1.642^∗^ROMA + 0.497^∗^Size))). The results showed that the AUC was 0.853 in the validation data (Figure 1(b), Table 2), demonstrating a good predictive efficiency. In addition, the DCA curve showed that the preoperative prediction of advanced endometrial cancer by the combination of ROMA index and tumor size benefited for almost all threshold probabilities not only in the training data (Figure 2(a)) but also in the validation data (Figure 2(b)). The above data exhibited that the combination of ROMA index and tumor size effectively predicted advanced endometrial cancer before surgery.

### 3.4. ROMA Index Was Also an Effective Predictive Factor for Advanced Endometrial Cancer before Surgery in the Training Data

In order to further select the more significant factors, we set *P* < 0.01 in forward stepwise regression, and then only ROMA index was left. Since ROMA index is convenient for screening, we continued to investigate whether only ROMA index was a preoperative predictor for advanced endometrial cancer. The results showed that the odds ratio of being positive ROMA index was 15.531 times that of negative value (Table 3). The above results demonstrated that ROMA index was also an effective predictive factor for advanced endometrial cancer before surgery in the training data.

### 3.5. ROMA Index Can Effectively Predict Advanced Endometrial Cancer before Surgery

**Figure 3:**
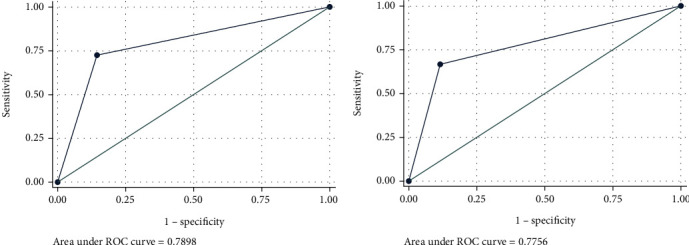
Receiver operating characteristic (ROC) curve for the preoperative prediction of advanced endometrial cancer by ROMA. (a) The area under ROC was 0.790 in the training dataset. (b) The area under ROC was 0.776 in the validating dataset.

**Figure 4:**
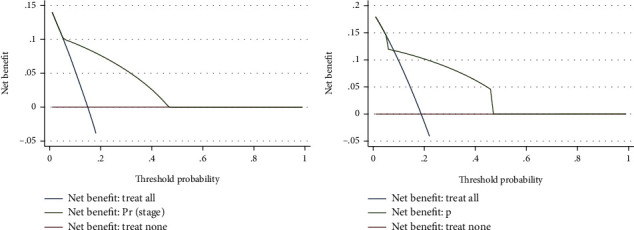
Decision curve analysis (DCA) curve of the prediction of advanced endometrial cancer before surgery by ROMA. (a) The attainable net benefit in the training dataset. (b) The attainable net benefit in the validating dataset.

AUC was also utilized to detect the predictive efficacy of ROMA index in the training data, and the results showed that the AUC was 0.790 (Figure 3(a), Table 3), indicating that only this biomarker could effectively predict advanced endometrial cancer before surgery. We further validated the predictive ability of ROMA index in the validation data. *P* value was generated using the following formula calculated from the training data, *P* = 1/(1 + exp(−(−2.876 + 2.743^∗^ROMA))). The results showed that the AUC was 0.776 in the validation data (Figure 3(b), Table 3), demonstrating a good predictive efficiency. Furthermore, the DCA curve showed that the prediction of endometrial cancer before surgery by ROMA index added more net benefits for almost all threshold probabilities not only in the training dataset (Figure 4(a)) but also in the validation dataset (Figure 4(b)). The above data demonstrated that ROMA index could effectively predict advanced endometrial cancer before surgery.

## 4. Discussion

About 89000 women die from endometrial cancer every year, while 380000 new cases are diagnosed annually throughout the world [15]. Although the majority of patients have a favorable prognosis, the mortality is increasing for endometrial cancer unlike other cancers even in the United States [16]. One of reasons is the poor prognosis of advanced endometrial cancer. The survival rate of patients with endometrial cancer beyond the uterus at diagnosis was only 5-15%, and it was related to half of all related deaths. Furthermore, when advanced endometrial cancer was not resectable, a median survival was only 2-8 months [6]. Therefore, the improvement of the prognosis of high-stage cancer could save a large number of patients since its high incidence rate.

Pretreatment evaluation and preoperative clinical staging are crucial for the management of endometrium cancer patients. However, it was proved that there was a high rate of inconformity between clinical staging and surgical-pathologic staging [17]. More importantly, 22.4% of patients were upstaged after surgical exploration and histopathological examination. Specifically, 8.6% of clinical stage I patients were upstaged to stage II, 11.8% to stage III, and 2.0% to stage IV, respectively. Therefore, the therapeutic schedule including the scope of operation for the underestimated patients may not be optimal. Therefore, accurate preoperative assessment of the stages is important for the treatment of advanced endometrial cancer. In clinical practice, preoperative imaging and serum tumor markers were applied. Transvaginal ultrasound is economic and mostly common, while the accuracy of assessing node metastasis still needs to be enhanced. Although large-scale medical equipments including MRI and PET-CT may have the highest efficacy, it is also hard for them to detect the microscopic node metastasis and is not practical in developing countries or at basic-level hospitals. Therefore, the reliable marker should be further investigated.

ROMA index is a scoring system that initially aims at distinguishing ovarian cancer from benign diseases via combination of CA125, HE4, and menopausal status [18, 19]. CA125, initially presented by Bast et al., is used as a tumor marker for adnexal masses [20]. Later, CA125 was also commonly applied for the detection of endometrial cancer, while CA125 has been proved to be elevated in 24.6% of EC patients and in only 10% of stage I and II patients [21]. Currently, HE4 was found to be a promising biomarkers for early detection, diagnosis, prognosis, and recurrence of ovarian and endometrial cancers [22]. HE4 seems to be less likely affected by benign pelvic masses, inflammation, endometriosis, adenomyosis, or pregnancy when compared to CA125. To further better predict the ovarian cancer, serum CA125, HE4, and ROMA index were compared in the diagnosis of epithelial ovarian cancer, and the results showed that ROMA had the best validity [23]. However, few studies were conducted to investigate the relationship between ROMA index and endometrial cancer. Here, we focus on the prediction of advanced endometrial cancer concerning its poor prognosis.

In this study, we not only included ROMA index but also involved other risk factors (age, menopause status, sterilization situation, parity, BMI, hypertension, diabetes, and tumor size) to investigate which one is the strongest predictor via multivariable logistic regression analysis. The results indicated that ROMA index and tumor size were the best subset of predictors for the probability of advanced endometrial cancer when *P* < 0.05 in the forward stepwise selection of logistic regression, and the AUC was as high as 0.884, which indicated that the combination of ROMA index and tumor size could effectively predict advanced endometrial cancer before surgery. Furthermore, when *P* was less than 0.01 in the forward stepwise selection of risk factors, only ROMA index was left as a predictor. The result showed that the odds ratio of being positive ROMA index was 15.531 times that of negative value, and the AUC was 0.790, indicating that only ROMA index was the predictive factor for advanced endometrial cancer before surgery. The DCA curve further mirrored that the prediction of endometrial cancer before surgery by ROMA index enhanced more net benefits for nearly all threshold probabilities. The results demonstrated that only ROMA index could effectively predict advanced endometrial cancer before surgery. However, limitations might exist in this study. Firstly, a prospective clinical control study should be further conducted. Secondly, the sample size was not large enough to further divide advanced endometrial cancer into stage IIIA, IIIB, IIIC, IVA, and IVB, and more patients should be included. Thirdly, this study was performed in one hospital, and more countries and areas should be involved.

## 5. Conclusion

In summary, ROMA index is an effective predictor for advanced endometrial cancer before surgery. Since ROMA index is a standard, measurable, and reliable laboratory test, it can be used as a reference tool for gynecologists to design the appropriate therapeutic schedule for patients with high-stage endometrial cancer before surgery.

## Figures and Tables

**Table 1:** Characteristics of patients in the training and validation datasets.

Characteristics	Training dataset			Validation dataset		
	Stage = 0 (*n* = 166)	Stage = 1 (*n* = 29)	*P*	Stage = 0 (*n* = 52)	Stage = 1 (*n* = 12)	*P*
Age, median (IQR)	55 (50, 61)	55 (51, 61)	0.93	54 (49, 58)	55.5 (50, 63)	0.65
Menopause no. (%)			0.40			0.74
No	55 (33.1)	12 (41.4)		15 (29)	4 (33)	
Yes	111 (66.9)	17 (58.6)		37 (71)	8 (67)	
Sterilization no. (%)			0.24			1.00
No	128 (77.1)	19 (65.5)		40 (77)	10 (83)	
Yes	38 (22.9)	10 (34.5)		12 (23)	2 (17)	
Parity, median (IQR)	1 (1, 2)	2 (1, 2)	0.66	1 (1, 2)	1 (1, 1.5)	0.59
BMI, median (IQR)	24.7 (22.6, 27.6)	24.9 (23.6, 27.7)	0.35	24.3 (22.3, 27.6)	22.5 (21.7, 25.2)	0.11
Hypertension no. (%)			0.83			0.27
No	108 (65.1)	20 (69.0)		38 (73)	11 (92)	
Yes	58 (34.9)	9 (31.0)		14 (27)	1 (8)	
DM no. (%)			0.38			0.31
No	143 (86.1)	27 (93.1)		48 (92)	10 (83)	
Yes	23 (13.9)	2 (6.9)		4 (8)	2 (17)	
Tumor size, median (IQR)	2.4 (1.4)	4.9 (2.2)	<0.001	2.7 (1.5)	5.5 (2.7)	<0.001
ROMA index no. (%)			<0.001			<0.001
No	142 (85.5)	8 (27.6)		46 (88)	4 (33)	
Yes	24 (14.5)	21 (72.4)		6 (12)	8 (67)	

Note: stage = 0, not advanced endometrial cancer; stage = 1, advanced endometrial cancer. Abbreviations: IQR: interquartile range; BMI: body mass index; DM: diabetes mellitus.

**Table 2:** Predictors for advanced endometrial cancer before surgery.

Intercept and variable	*β*	Odds ratio (95% CI)	*P*
Intercept	-4.098	—	—
ROMA	1.642	5.167 (1.673 to 15.957)	.004
Size	0.497	1.644 (1.211 to 2.231)	.001
Area under ROC curve		—	
Training dataset		0.884 (0.828 to 0.941)	
Validation dataset		0.853 (0.726 to 0.980)	

Note: *β*, regression coefficient; ROC: receiver operating characteristic.

**Table 3:** Prediction by ROMA index for advanced endometrial cancer before surgery.

Intercept and variable	*β*	Odds ratio (95% CI)	*P*
Intercept	-2.876	—	—
ROMA	2.743	15.531 (6.177 to 39.053)	.000
Area under ROC curve		—	
Training dataset		0.790 (0.63 to 0.77)	
Validation dataset		0.776 (0.630 to 0.922)	

Note: *β*, regression coefficient; ROC: Receiver operating characteristic.

## Data Availability

The data in this study are available upon reasonable request to the corresponding author.
